# Response to: “Are women equal? Considering impact of therapeutic abortion bans on science”

**DOI:** 10.1002/rth2.12754

**Published:** 2022-07-08

**Authors:** Fionnuala Ní Áinle, Jennifer Donnelly, Maeve Eogan

**Affiliations:** ^1^ School of Medicine University College Dublin (UCD) Dublin Ireland; ^2^ Department of Haematology Rotunda Hospital Dublin Ireland; ^3^ Department of Haematology Mater Misericordiae University Hospital Dublin Ireland; ^4^ Department of Obstetrics Rotunda Hospital Dublin Ireland; ^5^ Department of Obstetrics and Gynaecology Royal College of Surgeons in Ireland Dublin Ireland

Dear Professor Cushman,

We read with interest your insightful and powerful editorial^1^ describing the potential impact on women's lives and careers of the overturning of the *Roe v Wade* ruling in the United States.

As multidisciplinary care providers, we have seen at first hand the impact of very restrictive abortion legislation on women's health. The Eighth Amendment of the Constitution of Ireland was approved and signed into law following a referendum in 1983 (soon after the US *Roe v Wade* ruling). The Eighth Amendment conferred equal status to the right of life of the mother and the unborn, and what followed was 35 years of one of the most restrictive constitutional bans on therapeutic abortion in the world. The impact on women's health and status in society was profound.^2^, ^3^ Therapeutic abortion was permitted only when there was a real and substantial risk to life, as opposed to health, of the mother. In any other scenario, abortion was a criminal act. Throughout this time frame, many women in Ireland continued to access termination, either by traveling abroad (mainly to the United Kingdom) or by accessing abortion medicines online. Also throughout this time, Ireland was censured for a continuing breach of international human rights obligations: in the cases of *Mellet v Ireland* ^4^ and *Whelan v Ireland*,^5^ the United National Human Rights Committee found Ireland to be in violation of the International Covenant on Civil and Political Rights.^2^

This situation was particularly difficult because, as you have highlighted, unintended pregnancy is common. A recent study using data from country‐based surveys, official statistics, published studies, and data on live births from the World Population Prospects estimated that between 2015 and 2019, there were 121.0 million unintended pregnancies annually (80% uncertainty interval [UI], 112.8–131.5) in women aged between 15 and 49 years, with a global unintended pregnancy rate of 64 (UI, 60‐70) per 1000 women and 61% (UI, 58%‐63%) ending in abortion.^6^ Crucially, women living in countries where therapeutic abortion legislation was restricted still relied on abortion services, “potentially facing legal and physical risks for doing so.”^6^

It was therefore our privilege, as health care providers, to strongly support repeal of the Eighth Amendment to the Irish constitution in a civil society campaign termed “Together for Yes,” before a vote on May 25, 2018 (Figure 1). The campaign was successful, as evidenced by the overwhelming majority vote (66.4%) in favor. Legislation was enacted thereafter, permitting therapeutic abortion and care pathways were put in place to afford equitable access to therapeutic abortion in a range of contexts.^7^

**FIGURE 1:**
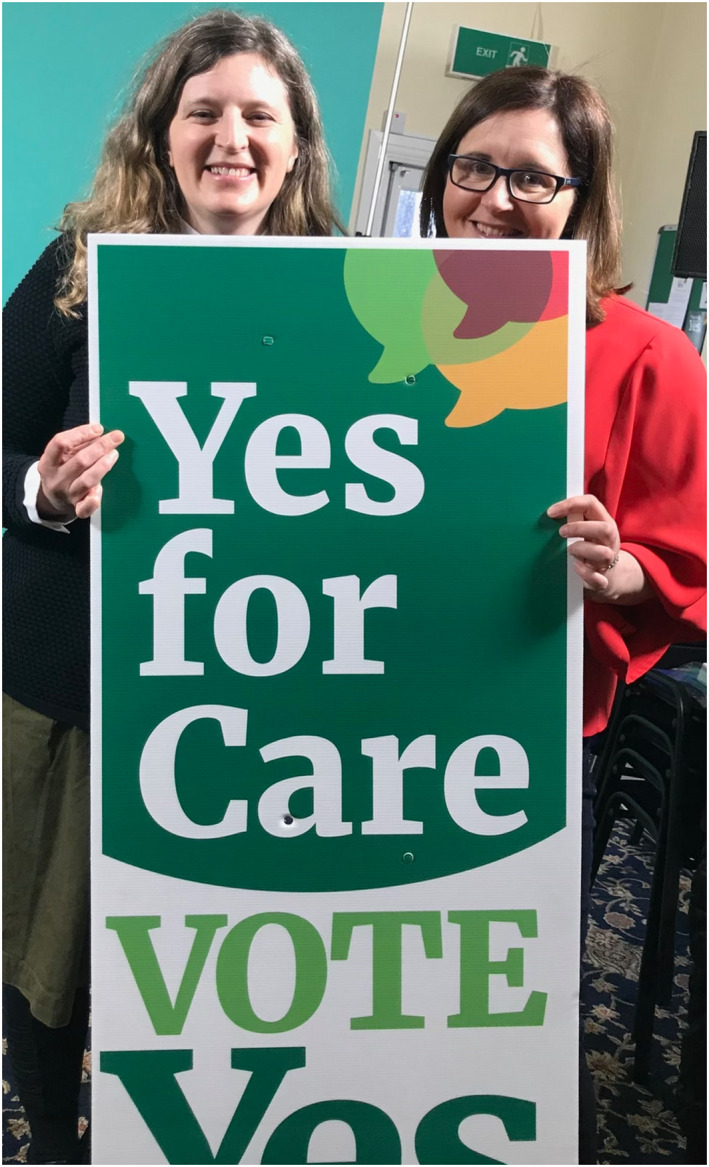
Irish Obstetricians Professors Donnelly (left) and Eogan (right), pictured during the successful “Together for Yes” campaign to repeal the Eighth Amendment to the Irish constitution in a 2018 referendum. The Eighth Amendment, imposed in 1983 (soon after the *Roe v Wade* ruling in the United States) had imposed a restrictive constitutional abortion ban in Ireland

Clinicians who cared for women with complex pregnancies and fatal fetal anomalies had a strong presence during the campaign and robustly advocated for Irish voters to consider the implications on health care of the Eighth Amendment. More than 80% of the entire Irish Institute of Obstetricians and Gynecologists supported repeal of the Eighth Amendment, including the masters (CEOs) of maternity hospitals, clinicians in sexual assault units, and the directors of several fetal‐maternal specialist centers—care providers who dealt on a weekly basis with the challenges imposed on safe and equitable health care provision by restrictive abortion legislation. The Irish Family Planning Association described the challenges and dangers faced by care providers and pregnant women: “The Eighth Amendment prevents doctors from acting in the best interests of their patients. In order to perform a lawful abortion, doctors must allow a woman’s condition to deteriorate until a risk to her health is unambiguously a risk to her life.”^3^ Professor Louise Kenny, an Irish professor of maternal and fetal health and now pro vice chancellor of the Faculty of Health and Life Sciences at the University of Liverpool, United Kingdom, stated, during her campaigning to repeal the Eighth Amendment: “In what is now 25 years of clinical practice, I have never met a woman who wanted a termination of pregnancy, but I have met many women who desperately needed one.”

The Eighth Amendment to the Irish constitution challenged the provision of high‐quality health care to pregnant women and posed a risk to lives.^8^ The practical reality was that thousands of women still accessed abortion care each year by traveling to other jurisdictions or illegally by way of procuring medications online. Both of these solutions carry their own risks, in terms of people accessing care without all necessary supports. Over the years, this situation affected many women with high‐risk complex pregnancy conditions or carrying a baby affected with a fatal fetal anomaly. It is impossible to truly know how many Irish women traveled to England to access abortion care or procured medications online. However, between 1980 and 2015, at least 165 438 women and girls in Ireland accessed UK abortion services.^3^ In 2016, 3265 women who underwent a therapeutic abortion in the United Kingdom gave an Irish address (a reduction from 2010, likely due to the more widespread availability of illegal and potentially unsafe abortion‐inducing medications online). A 2010 survey of sexual health behavior in women aged 18 to 45 reported that women with a low level of formal education were more likely to experience a crisis pregnancy compared with those with a higher education level (odds ratio, 2.29; 95% confidence interval [CI], 1.16‐4.52), suggesting a disproportionate effect on more vulnerable women.^9^ Few published data exist on the health impacts of unsafe abortion in Ireland. This is perhaps unsurprising, given the legal landscape before 2018.^10^

Lack of access to safe therapeutic abortion is a global health care issue that will now be faced by women and care providers in the United States: It is estimated that 22 million abortions continue to be performed unsafely each year, resulting in the death of up to ≈47 000 women annually and long‐term disability for another 5 million.^11^ The morbidities of unsafe abortion include hemorrhage, sepsis, genital injury, risk of chronic pelvic infection and infertility, and risks of blood transfusion in women of reproductive age.^11^, ^12^, ^13^, ^14^ In 2012, almost 7 million women were treated for complications of unsafe therapeutic abortion in the developing world.^15^ The economic and social consequences can be devastating: Data from the US Guttmacher Institute estimate that ≈8.3 million disability‐adjusted life years are lost annually because of the impact of unsafe abortion on women’s mortality and ill health.^16^ Moreover, unsafe abortion disproportionately affects women who are from disadvantaged and minority communities: The World Health Organization (WHO) reminds us that “in countries where induced abortion is legally highly restricted and/or unavailable, safe abortion has frequently become the privilege of the rich, while poor women have little choice but to resort to unsafe providers, causing deaths and morbidities that become the social and financial responsibility of the public health system.”^17^ The WHO therefore recommends that safe, legal abortion access is available to all women.^17^

Of direct relevance to the thrombosis and hemostasis community, it is particularly important to provide multidisciplinary input to women with higher‐risk pregnancies. In Ireland, before the repeal of the Eighth Amendment, this was highly relevant to women with, for example, acute venous thromboembolism (VTE) who wished to seek termination of pregnancy or women with cardiac conditions who were receiving anticoagulation. Both faced increased risks of bleeding and recurrent thrombosis, the mitigation of which was especially challenging before May 2018. This community needs no reminding that VTE and hemorrhage are leading causes of maternal morbidity and mortality.^18^ Countries with more permissive abortion legislation for termination of pregnancy tend to have lower rates of termination, and they tend to occur at earlier stages of pregnancy.^12^ This is clearly a much safer scenario, especially for women facing higher‐risk pregnancies or for those taking anticoagulant medications.

It has therefore been devastating to learn of the imminent overturning of the *Roe v Wade* ruling in the United States. In Ireland, we have for years admired the protection of women's rights enshrined in US law. Indeed, many countries globally look to the United States as a significant influencer of international health policy.^19^, ^20^, ^21^ The United States has a long track record of leadership in promoting excellent reproductive health and has been engaged in international research on family planning and population issues since the 1960s.^22^ Activities have been “designed to decrease the risk of unintended pregnancies and maternal and child mortality through effective interventions, including contraception, counselling, and post‐abortion care.”^22^ We stand with you and are grateful to you for your very strong appeal to consider the impact of this decision not only on women's lives and health but also on their careers and ability to participate as active members of the scientific community in the future. This is a deeply retrograde step for women's rights and health.

## AUTHOR CONTRIBUTIONS

FNA, JD and ME contributed equally to the concept and writing of this manuscript.

## RELATIONSHIP DISCLOSURE

None of the authors report a financial conflict of interest relevant to this work.
